# Change in mitochondrial capacity in elite triathletes, cyclists, and wrestlers over a training period of 28 days

**DOI:** 10.1007/s00421-025-05941-9

**Published:** 2025-08-27

**Authors:** Hannes Bossung, Christian Roth, Florian Netzer, Michael Behringer

**Affiliations:** 1 Department of Sports Medicine and Exercise Physiology, Institute of Sport Sciences, Goethe University, Frankfurt am Main, Germany; 2 Private Department for Venous Surgery, Munich, Germany

**Keywords:** Rapid-cuff inflation, Near-infrared spectroscopy, Elite athletes, Non-invasive assessment, Maximal oxygen consumption

## Abstract

**Purpose:**

This study investigated the change in mitochondrial capacity and *V*O_2_max in elite triathlon, cycling, and Greco-Roman wrestling athletes over a 28-day training period.

**Methods:**

Sixteen elite athletes (23 ± 2.5 years; 176 ± 6 cm; 76 ± 8 kg; 65 ± 6.9 ml/min/kg) participated. Mitochondrial capacity was assessed before (pre) and after (post) a 28-day training period by measuring the increase in deoxygenated hemoglobin (HHb) in the m. vastus lateralis during three consecutive one-minute rapid cuff occlusion periods using near-infrared spectroscopy. *V*O_2_max was measured via a treadmill ramp test at the same time points.

**Results:**

The analyses revealed significant differences between pre- and post-measurements, with significant improvements in the second (Δ*τ*2 = − 3.4 ± 2.7 s, *p* = 0.001) and third (Δ*τ*3 = − 5.0 ± 5.1 s, *p* = 0.006) occlusion period. Correlation analyses demonstrated a moderate negative relationship between the first occlusion tau (*τ*) rate and *V*O_2_max at pre-test (*r* = − 0.58, *p* = 0.02) and an even stronger negative correlation at post-test (*r* = − 0.62, *p* = 0.01). Within-subject analysis identified 14 athletes as responders.

**Conclusion:**

The changes in *τ* rates indicate significant improvements in mitochondrial capacity over a period of 28 days in elite athletes, underscoring the utility of NIRS-derived *τ* rates for monitoring changes in elite athletes.

**Graphical abstract:**

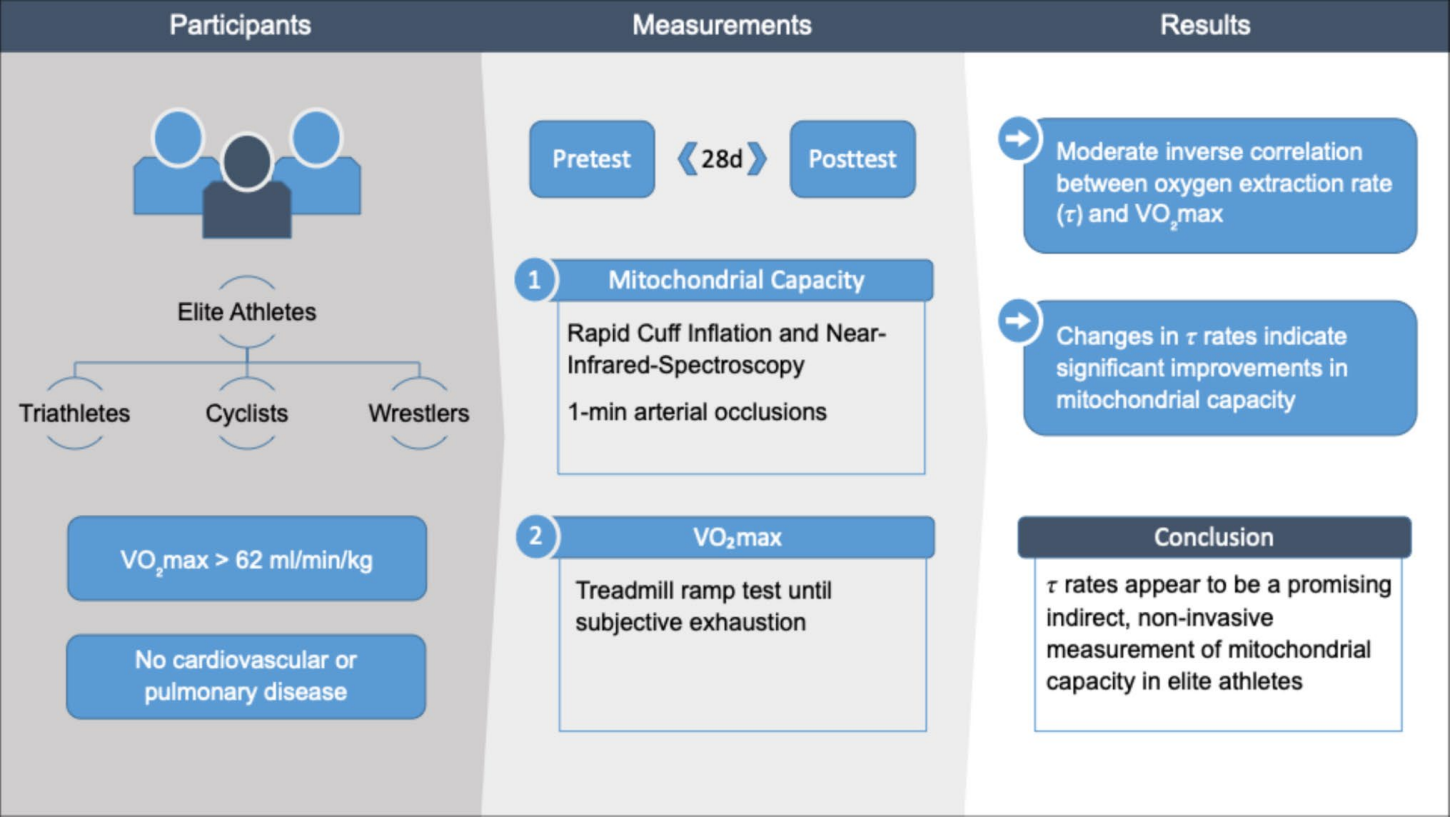

## Introduction

Mitochondrial capacity is widely recognized as a key determinant of athletic performance, particularly in endurance-based sports (MacInnis and Gibala 2017; Javadov et al. 2020). Mitochondria serve as the primary site of oxidative phosphorylation, enabling aerobic energy production that supports sustained physical exertion. Enhanced mitochondrial capacity facilitates more efficient oxygen utilization and substrate oxidation, resulting in increased ATP synthesis to meet the energetic demands of exercise. These adaptions are associated with advanced endurance (Granata et al. 2016), accelerated recovery between high-intensity bouts (Yamada et al. 2021), and superior performance in activities heavily reliant on aerobic metabolism (Al-Khelaifi et al. 2018). Moreover, augmented mitochondrial function can attenuate the onset of fatigue by mitigating the accumulation of metabolic by-products (e.g., lactate, hydrogen ions, and inorganic phosphate) and maintaining muscular contractile function under physiological stress (Allen et al. 2008). Despite the established importance of mitochondrial function, limited research has examined how pre-competition training influences mitochondrial capacity in elite athletes from diverse sporting disciplines. The present study addressed this gap by investigating changes in mitochondrial capacity among elite triathletes, cyclists, and wrestlers over a 28-day training period.

Near-infrared spectroscopy (NIRS) has emerged as a valuable non-invasive tool for assessing mitochondrial capacity in sports settings, providing results comparable to phosphocreatine recovery rate measurements (McCully et al. 2020; Hanna et al. 2021). By measuring changes in oxygenated and deoxygenated hemoglobin concentrations, NIRS provides a glimpse into muscle oxidative metabolism. In combination with the rapid cuff inflation (RCI) method, it offers a robust approach to quantify mitochondrial capacity by assessing the muscular response to controlled oxygen deprivation, and thereby providing important information for athletic performance (McCully et al. 2020; Adami and Rossiter 2018). In this process, the RCI is used to interrupt arterial blood flow for a certain period of time, creating a controlled environment that allows for assessing tissue oxygenation and deoxygenation kinetics. A key parameter derived from this approach is the time constant tau (*τ*), which quantifies the accumulation rate of deoxygenated hemoglobin (HHb) during arterial occlusion and serves as a proxy for mitochondrial oxygen consumption, providing valuable insights into oxidative capacity. A lower tau rate, indicative of a more rapid HHb accumulation, suggests enhanced mitochondrial capacity and a higher oxidative capacity (McMahon and Jenkins 2002). However, it is important to note that *τ* is an integrated parameter influenced not only by mitochondrial respiration but also by vascular oxygen delivery and intramuscular oxygen buffering. Interpretation of *τ* should consider the potential contributions of these additional physiological factors. Prior work validates NIRS-derived *τ* rates against phosphocreatine recovery kinetics and mitochondrial respirometry (Ryan et al. 2014) though they remain indirect proxies for mitochondrial oxidative capacity. The *τ* parameter integrates vascular oxygen delivery, mitochondrial respiration, and intramuscular oxygen buffering, and thus cannot fully disentangle mitochondrial-specific adaptations from systemic or hemodynamic factors. Nonetheless, its non-invasive nature and strong correlation with gold-standard measures support its utility for tracking training-induced metabolic changes in athletic populations (Zuccarelli et al. 2020). This study leveraged NIRS and RCI techniques to investigate changes in mitochondrial capacity and measured maximum rate of oxygen consumption (*V*O_2_max**)** among elite athletes from three distinct sports—triathlon, cycling, and Greco-Roman wrestling—over a 28-day training period. This research aimed to address a significant gap in the literature as no previous studies have systematically examined longitudinal changes in mitochondrial capacity across diverse high-performance sports using non-invasive techniques. By examining these physiological parameters over a month-long training period, this research sought to uncover sport-specific adaptations in mitochondrial capacity and their relationship to maximal oxygen uptake. We hypothesized that (1) mitochondrial capacity and *V*O_2_max increase during the 28-day training period, (2) there is a negative correlation between accumulation rate of deoxygenated hemoglobin (tau rate) and maximum rate of oxygen consumption attained during physical exertion over a training period of 28 days, and (3) mitochondrial capacity differs between sports.

## Materials and methods

This longitudinal study was conducted in cooperation with the Olympic Training Center Rhein-Neckar with measurements performed over a period spanning from April 22nd to May 28th, 2024. The male elite athletes visited the laboratory twice with 28 days in between. The testing procedure comprised two distinct phases, encompassing a total duration of approximately three hours: the measurement of mitochondrial capacity followed by the measurement of *V*O_2_max. The break between the two measurements was exactly two hours. A standardized snack, consisting of a protein bar (Powerbar True Organic, cocoa peanut flavor, Active Nutrition International GmbH, Munich, Germany), was distributed during the two-hour break between the two tests.

This study received ethics approval from the Institutional Ethical Review Committee of Frankfurt University, FB05, documented under approval number 2024-10 from March 13, 2024.

### Participants

At the time of measurement, all participants (wrestlers, triathletes, and cyclists), prepared for either the German Championship, the European Championship, the World Championship, and/or the Olympic Games 2024 in Paris, France (Table 1), following individualized training regimens supervised by their national teams. The 28-day observation period corresponded with the late preparatory phase for competition, during which athletes engaged in sport-specific, high-intensity, periodized training. For triathletes and cyclists, this phase primarily emphasized the enhancement of endurance power output, whereas wrestlers focused on anaerobic performance and weight management strategies.

**Table 1:** Participant demographics

Characteristics	Wrestling *n* = 12	Triathlon *n* = 2	Cycling *n* = 2
Age (years)	20 ± 4	24 ± 3	25 ± 2
Height (cm)	176 ± 6	180 ± 2	181 ± 1
Weight (kg)	78 ± 10.1	64 ± 2.3	64 ± 3.5
LATT (mm)^a^	4 ± 1	3 ± 1	4 ± 1

^a^Local adipose tissue thickness measured using a caliper

The participants were required to have a *V*O_2_max greater than 62 ml/min/kg, verifying a high level of aerobic fitness since this threshold is commonly associated with competitive endurance performance and reflects optimal physiological adaptations necessary for elite athletic capabilities (Smith et al. 2020). Due to sickness, two participants were not able to complete the post-test. Another two participants had to be excluded due to low *V*O_2_max parameters, leaving 16 elite athletes for statistical analyses.

All participants had to be current members of a national or Olympic sports team, maintaining an active athlete status at the time of measurement. Due to limited access to female athletes during the recruitment period, only male athletes were included in the study. Furthermore, eligibility was contingent upon the absence of any diagnosed cardiovascular or pulmonary diseases within the last 12 months. Participants were briefed on the risks, study procedures, and objectives prior to the study, and each signed a written consent form.

### Methodological framework, occlusion protocol, standardization

Continuous-wave NIRS devices cannot directly measure the precise concentrations of oxyhemoglobin, deoxyhemoglobin, and myoglobin. To enable comparison of NIRS signals across different participants and trials, an ischemic calibration is essential. This procedure entails the inflation of the pressure cuff above systolic pressure until the lowest point in the NIRS signal, known as the nadir, is observed. This record is then considered to represent the 0 percent oxygenation level. Following the release of the cuff, a hyperemic response is triggered, leading to the highest point in the NIRS signal, the zenith, which is then designated as the 100 percent oxygenation reference point for signal normalization (Lagerwaard et al. 2021). In order to study muscle oxygen capacity with NIRS, cuff inflation is used to interrupt O₂ supply to the monitored muscles to dissociate O₂ consumption from O₂ supply measured by NIRS (Hamaoka et al. 1996). This approach is similar to measuring phosphocreatine recovery rates (McCully et al. 2020).

Although brief occlusions, such as sequences of 5 × 5 s, 5 × 10 s and 5 × 15 s, or like the Mito-6-Protocol (Ryan et al. 2013), have been used in the literature for evaluating rapid mitochondrial responses (Hamaoka et al. 1996), occlusions lasting one minute enable a more thorough assessment of both the immediate and marginally delayed mitochondrial and muscular reactions to a state of reduced oxygen supply (Hamaoka et al. 1996; Ryan et al. 2013; Hanna et al. 2021; McCully et al. 2020). However, given that the perceived discomfort increases with occlusion time, we adapted the Mito-6 protocol. In our study, the occlusion protocol consisted of three one-minute occlusions interspersed with 30-s rest periods. This adjusted protocol, coupled with a final 2-min arterial occlusion for ischemic calibration, promised to furnish a comprehensive and insightful evaluation of local muscle oxygen capacity.

Noteworthy is also the fact that one-minute occlusion periods are short enough to minimize artifacts and physiological adjustments that might alter the hemodynamic status of the muscle, such as reactive hyperemia or the myogenic response (Young et al. 2020). In detail, the protocol consisted of three one-minute occlusion periods with the cuff being rapidly inflated to 250 mmHg with 30 s of rest in between followed by a two-minute resting period before performing a two-minute occlusion period for ischemic calibration. After calibration, the NIRS signals were monitored for another two minutes while at rest. Pre-occlusion metabolic rate is usually increased by electrical stimulation (40–80 V, 4 Hz) or short-duration exercise, with Ryan et al. (2013) demonstrating similar initial phosphocreatine re-synthesis rates for both methods. To increase the local metabolic rate of the m. vastus lateralis, 15 s of subjective maximal contractions (Hamaoka et al. 1996) were applied to the muscle, followed by the occlusion protocol. In this study, we used the Hokanson AG101 Inflator Air and Power Source (D. E. Hokanson, Inc, Bellevue, USA) in combination with the Hokanson E20 Rapid Cuff Inflation system with a Hokanson SC10LD cuff. NIRS signals were recorded by a PortaMon NIRS device (Artinis Medical Systems, Elst, the Netherlands) and observed simultaneously in the Software (OxySoft, Version 3.2.72). The PortaMon NIRS device measured at a sampling rate of 10 Hz with a Differential Pathlength Factor (DPF) of 4, which corrects for the scattering and absorption of light in tissue. These settings are also manufacturer recommendations.

Given the minimal presence of adipose tissue among participants, its potential impact on the NIRS signals was deemed negligible. In terms of environmental conditions, we standardized temperature and humidity as much as possible throughout the experimental sessions. Both the pre- and post-tests were conducted in the same controlled environment to maintain consistency. Additionally, participants were instructed to abstain from caffeine and other supplements for 24 h prior to the measurements. The local muscle area was shaved the day before testing to eliminate any bias from hair and erythema. To ensure the integrity of the measurements and prevent external light from interfering, a black piece of cloth was placed over the NIRS transmitter. Additionally, a stretchable bandage was utilized to secure the NIRS sensor at the thickest part of the muscle, which was determined through palpation. The bandage was tightly fastened to prevent the sensor from shifting, while avoiding excessive pressure on the surrounding tissue, as per McManus et al. (2018). To ensure consistent placement of the PortaMon device for both the pre- and post-tests, a mark was painted onto the skin following the pre-test.

### ***V***O_2_max determination

For measuring *V*O_2_max, a ramp test on a calibrated Woodway PPS MED treadmill (Woodway GmbH, Weil am Rhein, Germany) was performed. After a five-minute warm-up at eight kilometers per hour and a treadmill incline of 1.5 percent to simulate air resistance, the ramp test began. The speed remained constant at 11 km/h throughout the test. Starting at 1.5% every 60 s, the incline increased by one percent until the athlete experienced subjective exhaustion (Eike et al. 2021). The K5 wearable metabolic system from Cosmed (COSMED S.r.l., Albano Laziale, Italy) and a Cosmed heart rate monitor measured the metabolic and physiological changes throughout the ramp test. The system was calibrated each time before usage.

### Statistical analysis

A monoexponential model was utilized to analyze the changes in deoxygenated hemoglobin (HHb) concentration in response to arterial occlusion. The model is given by the equation

$${\mathrm{HHb}}=A+\left(B\times \left(1-{e}^{-\frac{t}{\tau }}\right)\right),$$

where:

- *A* represents the baseline HHb concentration prior to occlusion indicating the local starting level of HHb in the muscle tissue.
- *B* denotes the amplitude of change from the baseline HHb concentration reflecting the maximum deviation in HHb concentration due to occlusion.
- *e* is the base of the natural logarithm, approximately equal to 2.718, a constant value used in the exponential function.
- *t* is the time variable with *t* = 0 marking the onset of arterial occlusion.
- *τ* (tau) is the time constant (in seconds) of the exponential decay, a parameter that characterizes the rate at which HHb concentration approaches its new equilibrium post-occlusion. A smaller *τ* signifies a rapid adjustment to occlusion, while a larger *τ* indicates a more gradual response.

The exponential term ${e}^{-\frac{t}{\tau }}$ models the dynamic response of HHb concentration as a function of time, delineating how quickly it transitions from the baseline level A to a new equilibrium influenced by occlusion. The time constant *τ* describes the rate at which the concentration of HHb approaches its baseline in an exponential decay process.

The model was fitted to the dataset using nonlinear least squares optimization via the *nlsLM* function from the *minpack.lm* package in R. This optimization aims to minimize the residuals between the observed HHb concentration and the values predicted by the model, thus determining an optimal fit that accurately captures the observed HHb dynamics during arterial occlusion.

When analyzing the raw NIRS data, it has been observed that there is a spike in HHb values almost simultaneously with reperfusion. This could be attributed to the conversion of stagnant oxy-Hb to HHb, which is then promptly detected by the PortaMon device upon release, thereby triggering the spike. The spike appears to be greater after the two-minute ischemic calibration compared to the one-minute occlusions. To get valid *τ* rates, this spike had not been included in any of the calculations.

For statistical analyses, a general linear two-way repeated measures ANOVA (occlusion [3] × time [2]) with Bonferroni correction was performed. To check whether all assumptions have been met, a visual review of box plots, Shapiro–Wilk's test of normal distribution, and Levene's test for equality of variance were carried out as well as per Roth et al. (2023). Since a significant occlusion × time interaction was revealed, the simple main effects were examined separately using (i) repeated measures ANOVA (occlusion) and (ii) repeated measures ANOVA (time). Additionally, univariate ANOVA was conducted to compare variance between the sports. Pearson correlations were further calculated to examine the strength of relationship between the parameters *τ* and *V*O_2_max in both the pre- and post-tests. To further assess individual responses to training, we conducted an in-subject analysis by calculating the mean change in *τ* for each participant and classified athletes as responders or non-responders based on whether their *τ* change exceeded the smallest worthwhile change threshold (SWC = − 0.56 s) as determined by the distribution of individual differences. Only data from study finishers were included (per-protocol analysis). Statistical significance was based on an *α* = 0.05. Data are presented as mean (standard deviation), including effect sizes if adequate.

All calculations were done in R using RStudio (Version 2023.09.1+494).

## Results

The occlusion × time model revealed a significant [(*F*2,30) = 8.076, *p* = 0.005] change in tau rates over time. Across time points, the second and third tau rate changed significantly from pre to post, with − 1.06 s (SD = 2.52; *p* = 0.11) for *τ*1, -3.38 s (SD = 2.73; *p* < 0.001) for *τ*2, and -5.00 s (SD = 5.06; *p* = 0.001) for *τ*3, representing the first, second, and third cuff occlusion periods, respectively (Table 2). Furthermore, *V*O_2_max did not increase over the 28-day period (Δ = 0.17, SD = 2.83 ml/min/kg, *p* = 0.81).

**Table 2:** Listing of tau rates (in seconds) with mean and SD and *V*O_2_max (ml/min/kg) of the pre-test and post-test; data are illustrated as pre-test data | post-test data

	Dataset	1st *τ* rate	2nd *τ* rate	3rd *τ* rate	*τ* mean ± SD	*V*O_2_max
Wrestling	1	17 | 13	22 | 17	40 | 27	26.3 ± 12.1 | 19.0 ± 7.21	63.2 | 63.2
	2	16 | 11	22 | 16	29 | 25	22.3 ± 6.51 | 17.3 ± 7.09	62.1 | 64.8
	3	23 | 17	25 | 21	28 | 28	25.3 ± 2.56 | 22.0 ± 5.57	62.9 | 63.1
	4	22 | 20	24 | 22	29 | 28	25.0 ± 3.61 | 23.3 ± 4.16	64.3 | 62.1
	5	11 | 13	20 | 21	36 | 36	22.3 ± 12.7 | 23.3 ± 11.7	64.2 | 62.2
	6	12 | 12	22 | 20	34 | 28	22.7 ± 11.0 | 20.0 ± 8.0	64.5 | 64.5
	10	10 | 10	17 | 15	29 | 26	18.7 ± 9.61 | 17.0 ± 8.19	63.2 | 71.2
	11	13 | 12	17 | 16	29 | 27	19.7 ± 8.33 | 18.3 ± 7.77	64.1 | 64.1
	12	10 | 10	23 | 16	31 | 23	21.3 ± 10.6 | 16.3 ± 6.51	65.0 | 65.0
	14	15 | 14	21 | 18	42 | 31	26.0 ± 14.2 | 21.0 ± 8.89	64.3 | 64.3
	15	17 | 14	27 | 17	42 | 32	28.7 ± 12.6 | 21.0 ± 9.64	62.1 | 62.1
	16	16 | 14	23 | 19	41 | 26	26.7 ± 12.9 | 19.7 ± 6.03	63.1 | 63.1
					23.75 ± 3.04 | 19.85 ± 2.36	63.58 ± 0.95 | 64.14 ± 2.46
Triathlon	7	9 | 11	21 | 18	30 | 27	20.0 ± 10.5 | 18.7 ± 8.02	71.1 | 65.1
	8	10 | 10	21 | 19	27 | 26	19.3 ± 8.62 | 18.3 ± 8.02	71.9 | 71.9
					19.65 ± 0.49 | 18.5 0.28	71.5 ± 0.57 | 68.5 ± 4.81
Cycling	9	9 | 12	18 | 18	23 | 25	16.7 ± 7.09 | 18.3 ± 6.51	69.9 | 69.9
	13	11 | 11	22 | 18	34 | 29	22.3 ± 11.5 | 19.3 ± 9.07	64.8 | 66.8
					19.5 ± 3.96 | 18.8 ± 0.71	67.35 ± 3.61 | 68.35 ± 2.19

**Fig. 1:**
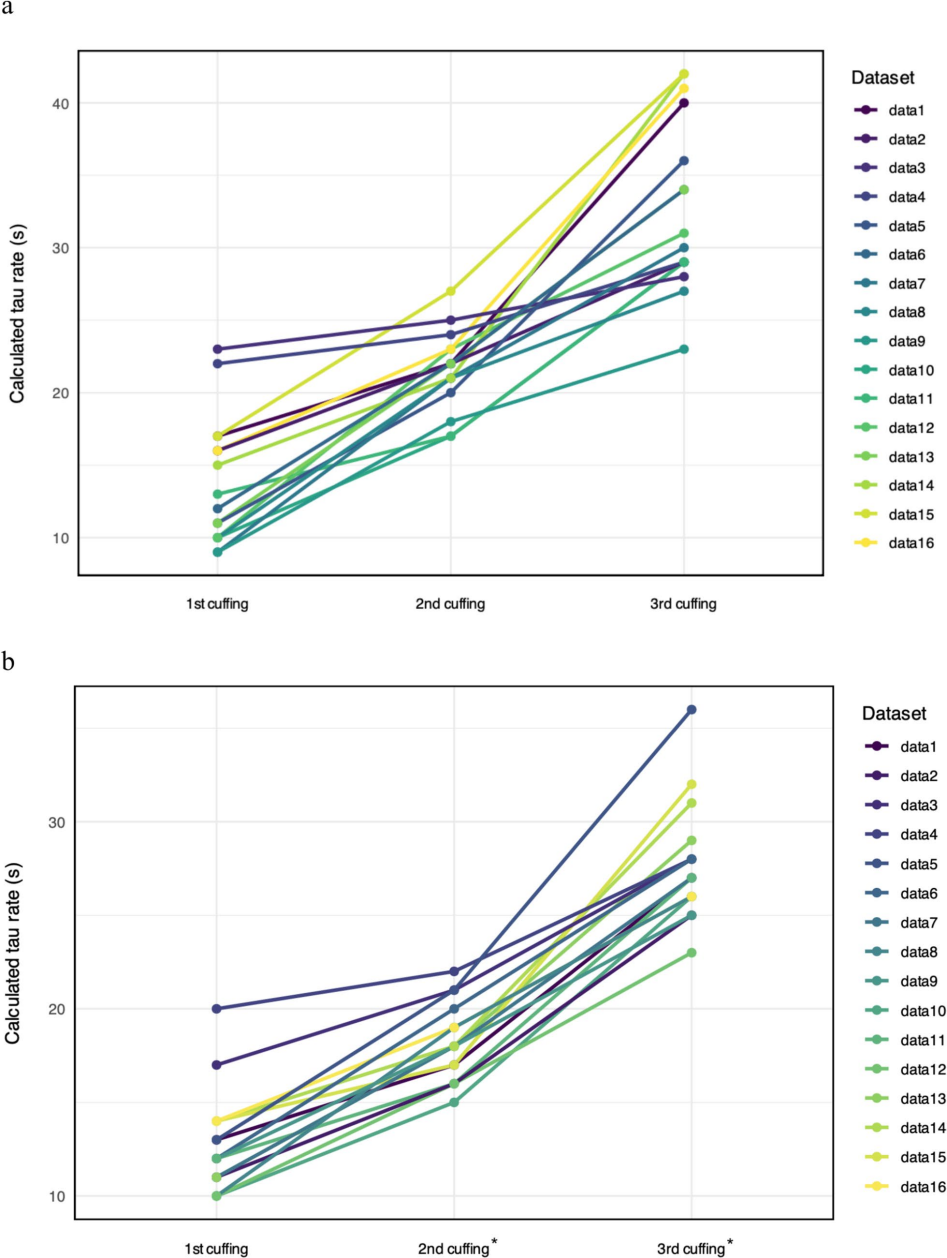
Development of tau rates (in seconds) across occlusions at pre- (**a**) and post-test (**b**). *Indicates statistically significant differences between pre- and post-tests

At the occlusion level, the calculated tau rates increased significantly between the first and the third occlusion, both at pre (*p* < 0.001) and post (*p* < 0.001; Fig. 1). In the pre-test, the distribution of *τ* rates across the three occlusion phases showed notable variability, with the first cuff occlusion exhibiting a mean *τ* rate of 13.81 s (SD = 4.4), the second cuff occlusion of 21.56 s (SD = 2.7; Δ*τ*2-1 = 7.75, SD = 3.42), and the third cuff occlusion of 32.75 s (SD = 5.9; Δ*τ*3-2 = 11.19, SD = 5.37).

Following the 28-day training period, the post-test results revealed a marked shift in *τ* distributions. The first cuff occlusion phase maintained a relatively stable mean *τ* rate of 12.75 s (SD = 2.7). The second cuff occlusion phase decreased with a mean *τ* rate of 18.19 s (SD = 2.0; Δ*τ*2-1 = 5.44, SD = 1.97). The third cuff occlusion phase decreased as well, with a mean *τ* rate of 27.75 s (SD = 3.1; Δ*τ*3-2 = 9.56, SD = 2.9 s).

**Fig. 2:**
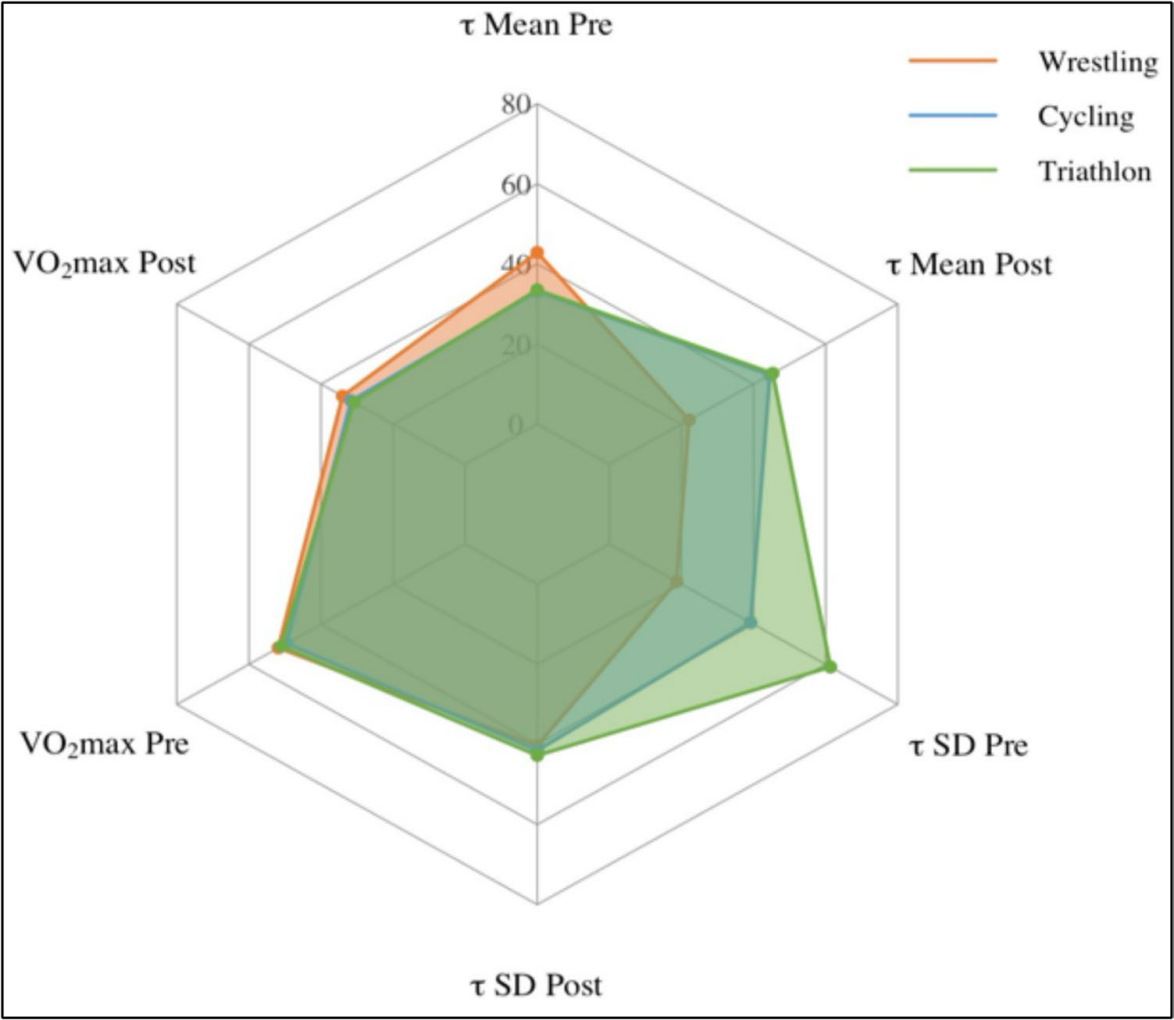
Group-wise comparison of *τ* mean, *τ* SD and *V*O_2_max at pre and post for wrestlers, cyclists and triathletes; for visual comparability, axes were scaled and values normalized

Triathletes demonstrated the most consistent profile across all parameters, with low *τ* mean and standard deviation values, as well as the highest *V*O_2_max values both at pre and post (Fig. 2). Cyclists showed slightly higher *τ* rates compared to triathletes but maintained a similar *V*O_2_max profile. In contrast, wrestlers exhibited the highest *τ* rates at pre and post. In wrestlers *τ*, rates improved modestly though remained higher than in the endurance-trained groups. Notably, improvements in *V*O_2_max were minimal and did not reach statistical significance in any of the groups (wrestling: 63.58 to 64.14 ml/min/kg, *t* = − 0.73, *p* = 0.48, triathlon: 71.5 to 68.5 ml/min/kg, *t* = 1.00, *p* = 0.50, cycling: 67.35 to 68.35 ml/min/kg, *t* = − 1.00, *p* = 0.50).

**Fig. 3:**
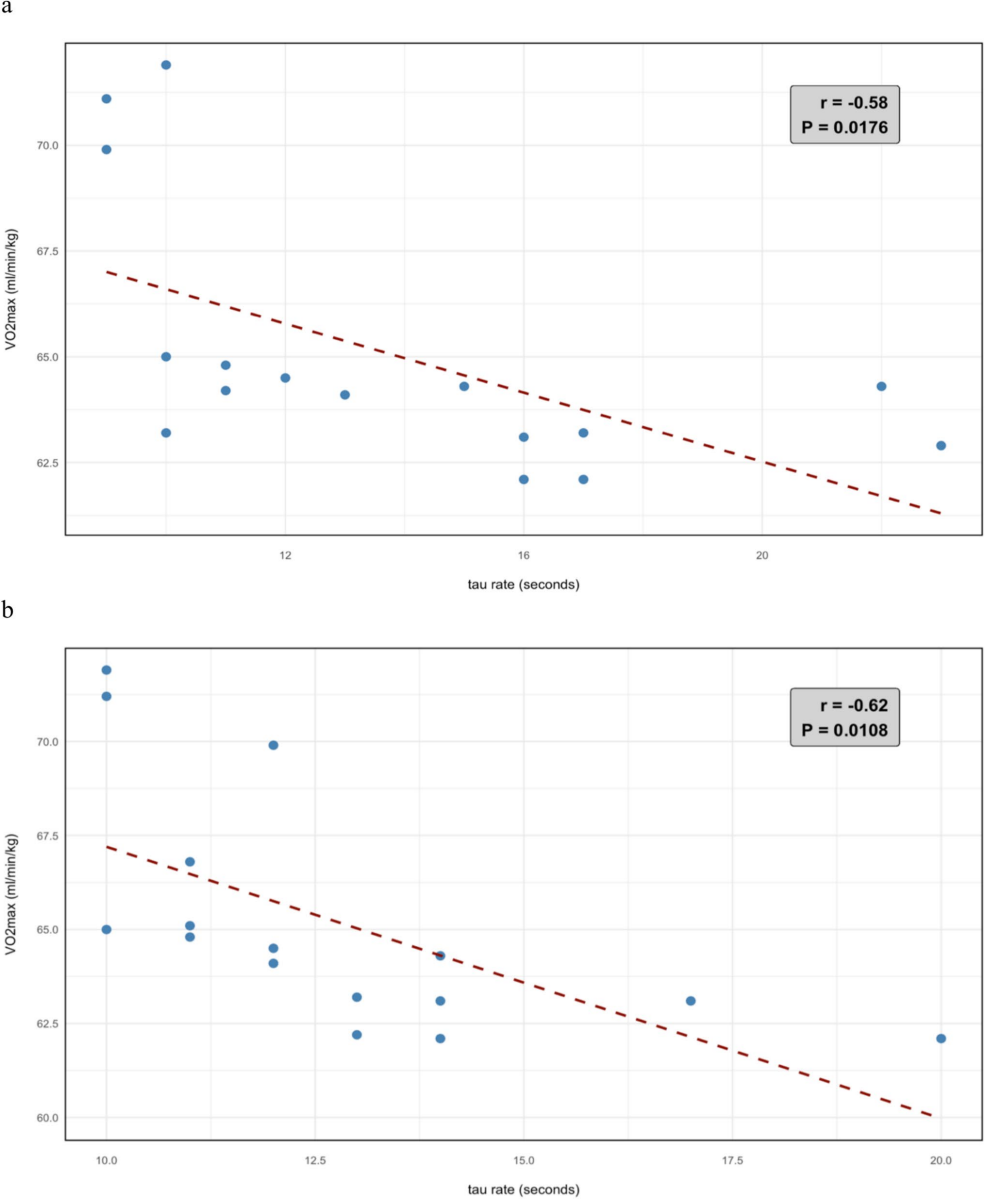
Correlation analysis of tau rate of first occlusion and *V*O_2_max of pre- (**a**) and post-test (**b**)

Our data revealed statistically significant moderate negative correlations between the lowest *τ* rates obtained during the first cuff occlusion phase and *V*O_2_max at pretest (*r* = − 0.58, *p* = 0.02) and at posttest (*r* = − 0.62, *p* = 0.01; Fig. 3).

**Fig. 4:**
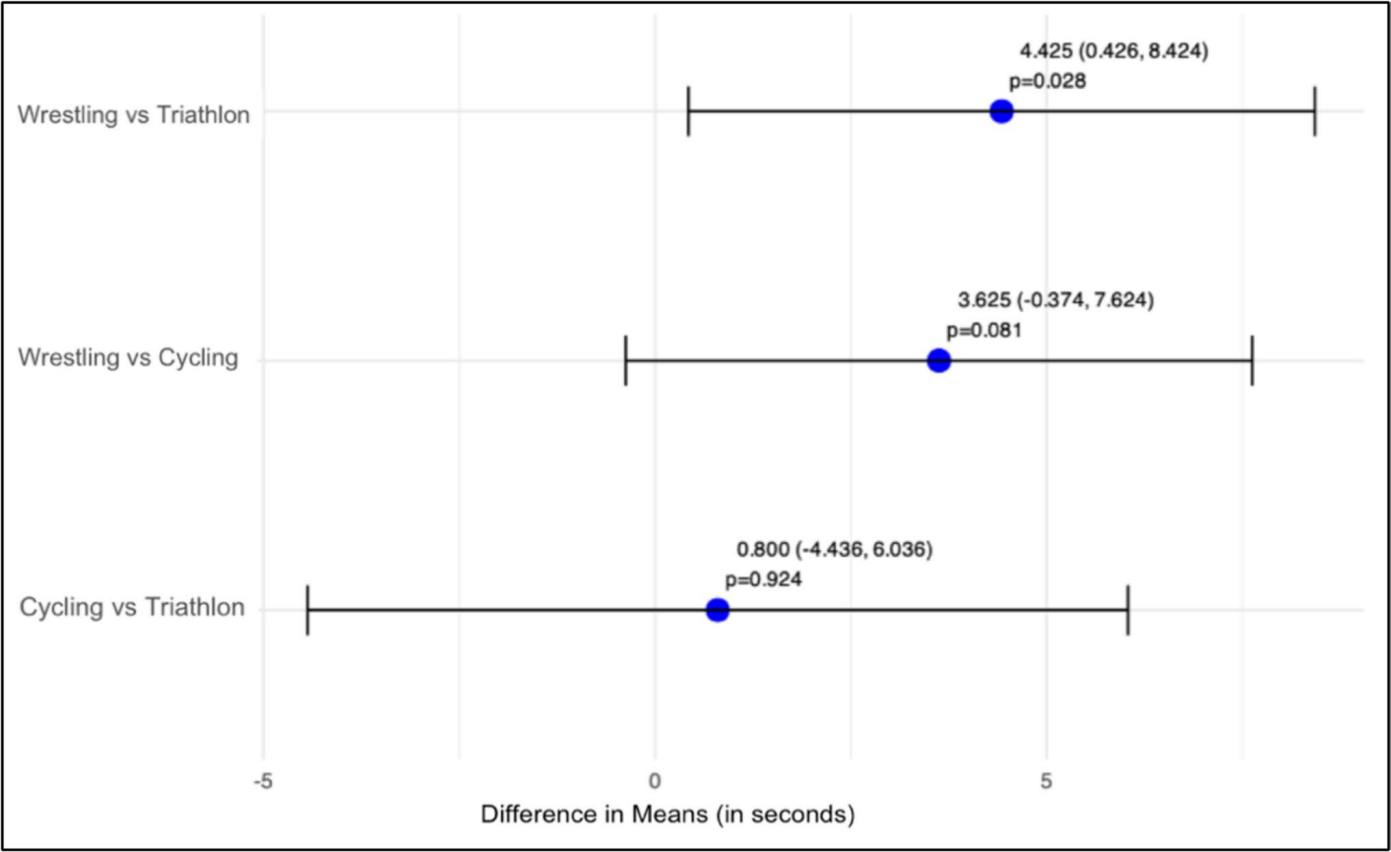
Pairwise post hoc comparisons of *τ* rates (in seconds) between disciplines with 95% confidence intervals

Comparisons among sports revealed that triathlon athletes had significantly lower mean tau rates compared to wrestlers (difference = 4.4 s, *p* = 0.028). This difference was also exposed between wrestling and cycling, although our model did not reach significance (difference = 3.6 s, *p* = 0.081). When comparing cycling and triathlon, no significant difference was found (difference = 0.8 s, *p* = 0.924) (Fig. 4).

**Fig. 5:**
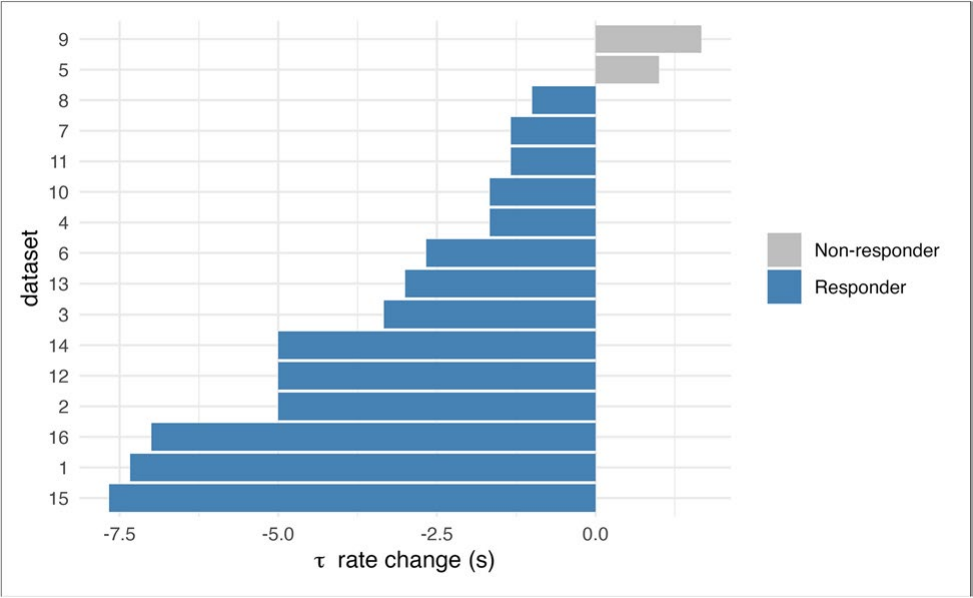
Individual Δ*τ* rates and responder classification based on SWC

Paired-sample *t* tests revealed a significant reduction in *τ* rates from pre to post (mean difference = − 3.02 s, *p* = 0.003), whereas changes in *V*O_2_max were not statistically significant (*p* = 0.128). To further distinguish meaningful adaptations, the smallest worthwhile change (SWC) in *τ* was calculated as − 0.56 s. Based on this threshold, 14 out of 16 participants were classified as responders, exhibiting physiologically relevant improvements in *τ* (Fig. 5). Only two individuals failed to surpass the SWC.

## Discussion

The primary goals of this study were to investigate changes in mitochondrial capacity over a 28-day training period across various sports of elite athletes, and second, to explore the correlation between mitochondrial capacity and maximum oxygen uptake. To the best of the authors’ knowledge, this study has been the first to apply the three one-minute occlusions protocol for determining the changes in HHb concentrations in a longitudinal design. We observed significant increases in the *τ* rate between occlusions at pre and post, significant improvements between the *τ* rate between pre and post, as well as indications of differences between sports. In-subject analysis showed a significant reduction in *τ* (Δ = − 3.02 s, *p* = 0.003), with 14 of 16 athletes classified as responders based on the smallest worthwhile change threshold. Correlation analyses exposed a moderate negative relationship between the first cuff occlusion *τ* rate and *V*O_2_max at the pre-test and an even stronger negative correlation at the post-test, thus supporting our second hypothesis. In light of these results, the discussed NIRS and RCI technique offers a robust framework for quantifying changes in mitochondrial capacity.

### Provision change of mitochondria over time

In the pretest, the *τ* rates across the three occlusion phases showed notable variability, with the first phase exhibiting the highest mitochondrial capacity and the third phase the lowest. After the 28-day training period, the posttest revealed significant improvements in mitochondrial capacity, particularly in the second and third occlusion phases, while the first phase remained relatively stable with consistent capacity. The significant improvement in mitochondrial capacity is in line with the results from the correlation analysis between *τ* rates and *V*O_2_max which revealed a moderate negative correlation between *V*O_2_max and mitochondrial capacity in the pretest. In particular, this metabolic relationship is consistent with recent findings of Mølmen et al. (2024) and implies that lower *τ* rates, which reflect higher mitochondrial capacity, are associated with higher *V*O_2_max levels, underscoring the role of efficient mitochondrial function in enhancing aerobic capacity. Aligning well with the findings of Giménez-Palomo et al. (2024), our data indicate the reciprocal relationship between mitochondrial capacity and *V*O_2_max. Moreover, our posttest correlation analysis revealed an even stronger negative correlation. This strengthened relationship suggests that the improvements in mitochondrial capacity, as evidenced by lower *τ* rates post intervention, have a more pronounced impact on *V*O_2_max. However, it should be noted that this moderate correlation may be influenced by the few athletes in our sample with exceptionally high *V*O_2_max values (around 70 ml/min/kg), rather than representing a consistent relationship across the entire cohort. Our findings demonstrate considerable variability in *τ* rates (10–15 s) among athletes with similar *V*O_2_max values (62–65 ml/min/kg), highlighting that oxygen uptake kinetics reflect distinct physiological mechanisms beyond maximal aerobic capacity. This variability may be explained by the fact that mitochondrial adaptations to training encompass not only changes in density, but also improvements in efficiency, enzyme content, cristae structure, and super-complex formation. Together, these adaptations can enhance oxidative capacity and accelerate *τ* rates, even in the absence of changes in *V*O_2_max (Lundby and Jacobs 2016; Lundby et al. 2017). While *V*O_2_max represents systemic oxygen transport and utilization limits, tau rate specifically captures the time constant for oxygen uptake adjustment during occlusion transitions. This kinetic parameter depends on mitochondrial respiratory capacity, oxidative enzyme activity, and muscle fiber composition, factors that can vary independently of *V*O_2_max (Christensen et al. 2016). Supporting this, Christensen et al. (2016) showed that just six high-intensity interval training sessions over two weeks improved *V*O_2_ kinetics (reduced *τ* rates) in tandem with enhanced mitochondrial electron transport system capacity and fatty acid oxidation despite no significant change in maximal oxidative enzyme activity. Notably, the lack of *V*O_2_max improvement despite faster *τ* rates aligns with evidence that mitochondrial functional adaptations (e.g., enhanced electron coupling efficiency, increased fatty acid oxidation) can occur independently of changes in maximal oxygen uptake (Lundby and Jacobs 2016). This dissociation between mitochondrial quality and traditional aerobic markers aligns with our observation of divergent *τ* rates in elite athletes with comparable *V*O_2_max.

### Sport-specific and individual *τ* rate responses

While sport-specific trends in *τ* rates were observed, interpretations should be made with caution due to an unbalanced number of athletes per sport. Triathlon athletes exhibited a narrow *τ* range with minimal variance between the pre–post measurements. This suggests a high degree of consistency and possibly a well-established training regimen that maintains mitochondrial capacity at a stable level. This consistency reflects the endurance nature of triathlon training, which continuously optimizes mitochondrial function through sustained aerobic exercise. The broader range in the first occlusion pre-test *τ* rates in cycling athletes suggests a higher variability in the mitochondrial capacity compared to triathlon athletes. However, similar to triathlon, the endurance-focused training results in relatively low *τ* rates overall, highlighting the homogeneous metabolic efficiency among the cyclists (Weiss et al. 2022). Wrestlers, in contrast, displayed the greatest heterogeneity in *τ* rates, consistent with the sport’s mixed acyclic, anaerobic bursts with aerobic components (Manojlovic et al. 2022). Despite high inter-individual variation, this group showed a significant improvement in *τ* rates compared to triathletes (Δ = 4.4 s, *p* = 0.028, Fig. 4), as hypothesized in our third hypothesis, though no significant difference emerged in other group comparisons. Potential differences between sports should be interpreted with caution given its exploratory nature. To address this limitation, we conducted a within-subject analysis using the smallest worthwhile change (SWS = − 0.56 s) to classify responders. Fourteen of 16 participants exceeded this threshold, suggesting mitochondrial capacity changes regardless of sport. Only two athletes were classified as non-responders (Fig. 5).

Overall, the analysis of *τ* rates across these sports underscores the differential impacts of specific training regimens on mitochondrial oxidative capacity, consistent with the results of Al-Khelaifi et al. (2018). When considering *V*O_2_max values, it is important to note that the two triathletes, followed by the two cyclists, exhibited the highest *V*O_2_max levels in this study. It is noteworthy that the overall *τ* rates improved across all three sports over the 28-day training period, indicating enhanced aerobic system capacity in each group, although *V*O_2_max did not improve, partially confirming our first hypothesis regarding mitochondrial adaptations but not systemic changes.

### Physiological determinants of reoxygenation kinetics and sport-specific adaptations

Key factors influencing mitochondrial capacity kinetics include capillary density, mitochondrial respiratory capacity, myoglobin content, and muscle fiber-type distribution. In particular, individual variations in capillary density can significantly impact *τ* variability, as a higher capillary-to-fiber ratio enhances the efficiency of oxygen delivery to muscle fibers and enables faster re-oxygenation regardless of mitochondrial function. This influence of capillary density may help account for the considerable differences in *τ* rates observed among athletes with similar *V*O_2_max values (Fig. 3a), suggesting that those with greater capillarization can achieve more rapid muscle oxygen kinetics even when their maximal aerobic capacity is comparable to others in the group. A higher capillary-to-fiber ratio reduces oxygen diffusion heterogeneity, accelerating re-oxygenation by minimizing transit time from erythrocytes to mitochondria, while enhanced mitochondrial respiratory capacity, reflected in lower *τ* rates, increases oxygen consumption velocity, rapidly restoring intramuscular oxygen reserves (Bosutti et al. 2015). Myoglobin buffers transient hypoxia and modulates re-oxygenation kinetics (Adepu et al. 2024) and fiber-type composition further shapes these dynamics as Type I fibers exhibit faster re-oxygenation due to elevated myoglobin levels and oxidative enzyme density compared to Type II fibers (Zuccarelli et al. 2020). These mechanisms are selectively optimized through sport-specific training: endurance athletes (triathletes/cyclists) exhibit higher capillary density and Type I fiber oxidative capacity, which streamline oxygen diffusion and utilization (Shadiow et al. 2023), whereas wrestlers’ anaerobic–hypertrophic training prioritizes mitochondrial biogenesis in Type II fibers. Despite lower capillarization, wrestlers’ intermittent hypoxic training may enhance mitochondrial respiratory control in Type II fibers, compensating via improved oxygen extraction efficiency (Zuccarelli et al. 2020). This divergence explains the broader *τ* variability in wrestlers as Type II fibers inherently exhibit reduced oxidative enzyme activity (e.g., citrate synthase) and capillary-to-fiber ratios (Bishop et al. 2019). Collectively, *τ* rates integrate these vascular and mitochondrial adaptations, serving as a more sensitive biomarker of sport-specific metabolic remodeling than *V*O_2_max, which reflects systemic oxygen delivery limits rather than localized functional optimization.

### Limitations

Although this study was carefully planned, it is not free of limitations. One is the decision not to correct for blood volume changes (BVC) in the NIRS measurements. It is known that pressure can induce minor shifts within the vascular and interstitial spaces, potentially affecting NIRS signals (Ryan et al. 2012), but the literature lacks precise guidelines on pressure thresholds and durations required BVC. While preliminary data showed stable total hemoglobin (tHb) during occlusions (± 0.01 g/dl), future studies in populations with greater tHb variability should consider BVC adjustments. Second, the absence of cardiovascular parameters (e.g., stroke volume, cardiac output) limits our ability to disentangle central (oxygen delivery) and peripheral (mitochondrial) determinants of *τ* rate adaptations. NIRS-derived *τ* rates provide a composite measure of mitochondrial function across subsarcolemmal versus intermyofibrillar mitochondrial subpopulations. While this limits resolution of fiber-type-specific adaptations, it aligns with our aim to assess global mitochondrial capacity changes in elite athletes. Future work should integrate histology or imaging to delineate contributions from subsarcolemmal and intermyofibrillar mitochondria.

Although hampering external validity, this study did not include female participants, justified by methodological pilot goals and evidence of no significant sex differences (Junker et al. 2022). Recruitment difficulties for elite female athletes further constrained the feasibility of including both sexes in this pilot study.

Training load metrics were not quantified due to logistical constraints inherent in elite athlete preparation environments. Additionally, the small sample sizes in the triathlon (*n* = 2) and cycling (*n* = 2) groups necessitate cautious interpretation of between-sport differences and underscore the importance of future investigations with larger cohorts with a broader *V*O_2_max range to validate these preliminary findings. Results should be viewed as preliminary trends rather than definitive group effects.

Ultimately, occasional leg movements, despite strict instructions, may have introduced signal variability in NIRS data. While likely minor, such motion artifacts remain a technical limitation and should be mitigated in future studies through more rigorous stabilization protocols.

## Concluding remarks and perspective

This study demonstrated that mitochondrial capacity, represented by *τ* rates, showed measurable changes within a 28-day training period in elite athletes. Previous research has demonstrated good to excellent test–retest reliability for NIRS-derived *τ* measurements, with intra-class correlation coefficients (ICCs) ranging from 0.82 (Hanna et al. 2021) to 0.93 (Ryan et al. 2013) across repeated testing sessions. Given the potential of non-invasive NIRS and RCI techniques to assess mitochondrial capacity (McCully et al. 2020), the progressive increase in *τ* rates and their variability across the three arterial occlusion phases indicate a robust adaptive response. The lack of direct biochemical markers precludes definitive conclusions regarding specific mitochondrial adaptations; the observed changes in *τ* nonetheless provide valuable insights into muscle oxygen kinetics metabolic adaptations. The moderate inverse correlation between *τ* rates and *V*O_2_max suggests that *τ* kinetics may reflect mitochondrial functional adaptations. However, further validation against direct measures of mitochondrial capacity (e.g., respirometry) is required to confirm *τ*’s reliability as a standalone biomarker. These findings reinforce the notion that mitochondrial adaptations can occur independently of systemic markers like *V*O_2_max as observed in elite athletes with cardiovascular ceilings (Lundby and Jacobs 2016). Future research should extend the 28-day intervention period and include larger, sex-balanced cohorts with diverse *V*O_2_max levels to clarify *τ*’s relationship to mitochondrial capacity across multidisciplinary populations. Finally, a more granular exploration of individual sports could elucidate sport-specific metabolic demands and uncover deeper, individualized responses. Such insights may not only optimize performance in elite athletes but also hold broader applications for endurance training methodologies in diverse populations.

## Data Availability

The datasets generated during and/or analyzed during the current study are available from the corresponding author on reasonable request.

## Abbreviations

ANOVA: Analysis of variance
ATP: Adenosine triphosphate
BVC: Blood volume changes
DPF: Differential pathlength factor
HHb: Deoxygenated hemoglobin
ICC: Intra-class correlation coefficient
LATT: Local adipose tissue thickness
NIRS: Near-infrared spectroscopy
O₂Hb: Oxygenated hemoglobin
RCI: Rapid cuff inflation
SWC: Smallest worthwhile change
*τ*: Tau rate
tHb: Total hemoglobin
*V*O_2_max: Maximum rate of oxygen consumption
